# Starch–Mucilage Composite Films: An Inclusive on Physicochemical and Biological Perspective

**DOI:** 10.3390/polym13162588

**Published:** 2021-08-04

**Authors:** Mansuri M. Tosif, Agnieszka Najda, Aarti Bains, Grażyna Zawiślak, Grzegorz Maj, Prince Chawla

**Affiliations:** 1 Department of Food Technology and Nutrition, Lovely Professional University, Jalandhar 144411, Punjab, India; tosifmansuri444@gmail.com; 2 Department of Vegetable Crops and Medicinal Plants, University of Life Science in Lublin, Doświadczalna Street 51A, 20-280 Lublin, Poland; grazyna.zawislak@up.lublin.pl; 3 Department of Biotechnology, Chandigarh Group of Colleges, Landran, Mohali 140307, Punjab, India; aarti05888@gmail.com; 4 Department of Power Engineering and Transportation, University of Life Sciences in Lublin, Gleboka 28, 20-612 Lublin, Poland; grzegorz.maj@up.lublin.pl

**Keywords:** starch, mucilage, biodegradable film, food packaging

## Abstract

In recent years, scientists have focused on research to replace petroleum-based components plastics, in an eco-friendly and cost-effective manner, with plant-derived biopolymers offering suitable mechanical properties. Moreover, due to high environmental pollution, global warming, and the foreseen shortage of oil supplies, the quest for the formulation of biobased, non-toxic, biocompatible, and biodegradable polymer films is still emerging. Several biopolymers from varied natural resources such as starch, cellulose, gums, agar, milk, cereal, and legume proteins have been used as eco-friendly packaging materials for the substitute of non-biodegradable petroleum-based plastic-based packaging materials. Among all biopolymers, starch is an edible carbohydrate complex, composed of a linear polymer, amylose, and amylopectin. They have usually been considered as a favorite choice of material for food packaging applications due to their excellent forming ability, low cost, and environmental compatibility. Although the film prepared from bio-polymer materials improves the shelf life of commodities by protecting them against interior and exterior factors, suitable barrier properties are impossible to attain with single polymeric packaging material. Therefore, the properties of edible films can be modified based on the hydrophobic–hydrophilic qualities of biomolecules. Certain chemical modifications of starch have been performed; however, the chemical residues may impart toxicity in the food commodity. Therefore, in such cases, several plant-derived polymeric combinations could be used as an effective binary blend of the polymer to improve the mechanical and barrier properties of packaging film. Recently, scientists have shown their great interest in underutilized plant-derived mucilage to synthesize biodegradable packaging material with desirable properties. Mucilage has a great potential to produce a stable polymeric network that confines starch granules that delay the release of amylose, improving the mechanical property of films. Therefore, the proposed review article is emphasized on the utilization of a blend of source and plant-derived mucilage for the synthesis of biodegradable packaging film. Herein, the synthesis process, characterization, mechanical properties, functional properties, and application of starch and mucilage-based film are discussed in detail.

## 1. Introduction

Over the past few years, non-degradable plastic-based materials have created a major challenge for the whole world due to their excessive use in the industrial and domestic sectors and global production which exceeds 400 Mt/year [1]. Synthetic food packaging materials are considered highly harmful to the environment (soil, water, and air) [2]. They are also involved in the human food chain, causing several diseases such as skin diseases, DNA damage, oxidative injury, asthma, infertility, and cardiovascular disease due to their negligible decomposition ability [3]. Researchers have frequently been looking for novel biodegradable materials to address the problem of plastic waste disposal [4]. Therefore, natural biopolymers such as plant or animal-derived proteins and polysaccharides (starch, gums, mucilage, cellulose, albumin, and gelatin), microorganism-derived bio-polyesters (PHAs; polyhydroxyalkanoates), and biotechnology-derived bio-polyesters (PLA; polylactic acid) have been explored as potential alternatives for conventional plastics [5,6]. Moreover, the film prepared from plant-derived bio-polymers improves the shelf life of commodities by protecting them against interior and exterior factors such as microorganisms, moisture, gases, and temperature. Moreover, edible films can also be used as carriers of ingredients and components such as vitamins, minerals, antioxidants, antimicrobials, and nutraceuticals, in addition to acting as a barrier [7]. All of the above factors are impossible to attain with single polymeric packaging material. Therefore, the properties of edible films can be modified based on the hydrophobic–hydrophilic properties of bio-polymer [8]. In such cases, several plant-derived combinations can be used as an effective binary polymer such as protein–protein, protein–carbohydrates, and carbohydrates–carbohydrates. Furthermore, hydrophilic polymers are famous due to their good mechanical properties while hydrophobic polymers have excellent moisture barrier properties [9]. Generally, starch is an edible carbohydrate complex, composed of a linear polymer, amylose (a linear molecule with few branches), and amylopectin (branched-chain molecule). Therefore, the presence of amylose in large quantities provides excellent strength while a high level of amylopectin is responsible for the reduction of the tensile strength during the production of a film [10]. They have usually been considered as a favorite choice of material for food packaging applications due to their excellent forming ability, low cost, and environmental compatibility. Naturally, starch is a water-insoluble and semi-crystalline component that is widely extracted from various plants, foods, and agricultural waste [11]. The characteristics of starch-based films vary abundantly depending on their synthesis procedures and plant origin. Furthermore, starch is generally modified to improve the functional properties and provide good flexibility or versatility of films for food packaging applications [12]. However, starch-based films have limitations in their ability to bear various environmental factors such as temperature, pressure, and natural gases during the handling due to their low strength, flexibility, rigidity, and high hydrophilic nature [9]. Starch films are brittle and lack mechanical integrity, therefore several studies have been conducted on starch modifications and the addition of modifiers to the film matrix to improve the barrier, thermal, and mechanical properties of the starch films. Thus, low moisture resistance, lower mechanical properties, and release of low molecular weight plasticizer from the starch matrix are all disadvantages that were observed by Zhang et al. [13]. To overcome this issue, the combination of starch and mucilage can be used as a binary polymer alternative to improve the mechanical properties of the packaging film. Additionally, the addition of several biopolymers such as cellulose, gum, and gelatin into a starch blend can change the network formation in the film matrix, improving the physicochemical and biological properties of the film [14]. Moreover, mucilage is a water-soluble edible polysaccharide, extensively used in the food industry due to its excellent functional properties (antimicrobial, antioxidant, water-holding, oil holding, and foaming capacity), and diverse industrial applications such as thickening agent, binding agent, emulsifying agent, and suspending agent [15]. Mucilage has a great potential to produce a stable polymeric network that confines the starch granules, which delay the release of amylose in resulting the improvement of the mechanical property of films [16]. Moreover, mucilage can be used as an effective ingredient for the formulation of sustainable, cost-effective, eco-friendly products [17]. However, very few reports have been published on starch and mucilage-based composite film. Therefore, in this review, we summarize the physicochemical and biological properties of the starch–mucilage-based film. Additionally, the application of starch–mucilage-based film in the food industry is discussed with a schematic diagram and mechanism.

## 2. Synthesis of Starch–Mucilage Composite Films

**Figure 1:**
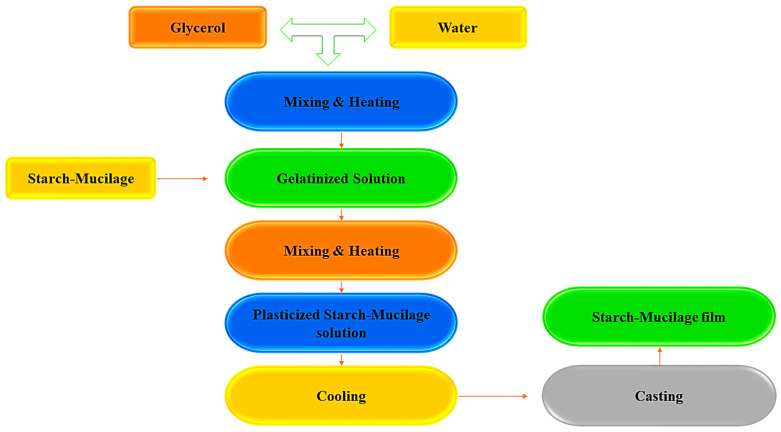
Synthesis of the starch–mucilage film by casting method.

Generally, biopolymer (starch–mucilage)-based edible film can be synthesized by two methods: the casting and extrusion techniques, also known as wet and dry methods [18]. Moreover, the solubility of starch–mucilage and other additives is an important factor for the casting method of film formation, while thermo-plasticity of starch–mucilage along with gelatinization characteristics, glass transition, and phase transitions can be recognized for the extrusion method [19]. Among these two methods, the casting method is widely used for the synthesis of starch–mucilage films due to its low production cost and is also known as the solvent-casting method. Furthermore, the solvent casting method comprises three successive steps to prepare a film from the binary polymers (starch and mucilage): (i) solubilization of starch–mucilage sample in an appropriate solvent, (ii) casting or forming of the prepared starch–mucilage solution in molds, and (iii) drying of starch–mucilage-casted solution [20,21]. The synthesis of the starch–mucilage film is explained in Figure 1.

Glycerol is most commonly used to dissolve or disperse the starch and mucilage polymers; this process is known as solubilization. The resulting solution is poured into a specified mold or glass plate during the casting process. The drying process allows the solvent to evaporate, resulting in a starch–mucilage film that binds to the mold [22]. For the casting of films, drying techniques such as vacuum driers, microwaves, tray dryers, and hot air ovens are used to evaporate the solvents and peel the film [23]. In this context, films were prepared from the *Plantago psyllium* starch and seed mucilage by Krystyjan et al. [24]. In their study, they proved that films prepared with binary polymers have strong physicochemical properties. Additionally, the binary combination of starch and mucilage showed high thermal stability and reduced the decomposition process as compared to film prepared by single polymer starch only. Moreover, the amount of pseudo-plasticity was decreased with the increase in the starch concentration due to galactomannans structure being composed of a mannose backbone, which interchanges with galactose chains. The breakdown of the network induced by shear forces was faster than the recovery of the structure in systems where thixotropy characteristics were dominant. Weak physical bonds were broken as a result of shear forces, and the interior structure disintegrated into separate particles. Additionally, with the addition of mucilage, the rheological behavior of film-forming solution showed various changes such as increased shear stress, higher consistency coefficient, and influence on the value of the area of the hysteresis loop [17].

**Figure 2:**
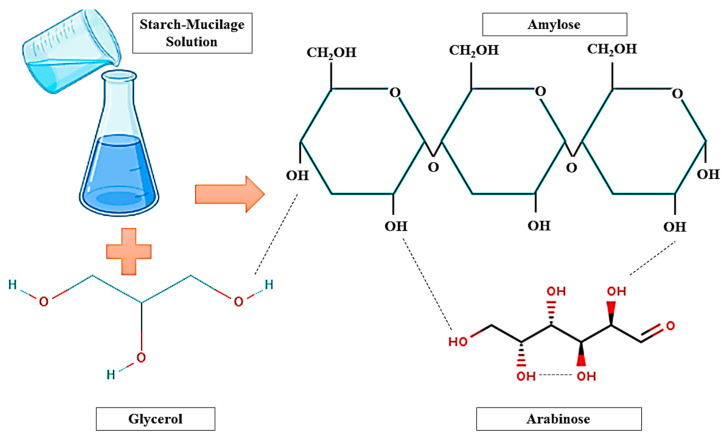
Interactions between starch–mucilage and glycerol through hydrogen bondings.

Consequently, plasticizers and cohesive matrix can be used for the development of easily peeled starch–mucilage edible films with an excellent uniform microstructure, thermal stability, barrier properties, and mechanical properties. The starch–mucilage pre-fabricated film can be used in several industrial applications, mainly in food applications. Here, the film can act as a good barrier, protector, and reduce the loss of water from food products [21]. Therefore, they can enhance the shelf-life of food products. The thickness of the film can be decreased by increasing the concentration of starch nanocrystal due to the excellent composite formation between starch nanocrystals and mucilage [3]. In addition, the thermal property, mechanical strength, and barrier property of starch–mucilage edible film can be improved with the increasing concentration of plasticizers. However, the casting method requires more drying time, which is the main drawback of this method. Likewise, edible films prepared from the potato husk starch and prickly pear peel Mucilage showed a positive impact on their properties such as excellent flexibility, transparency, and bright appearance. The water solubility of the film was influenced by the potato husk starch content and a high amount of glycerine and prickly pear peel leads to films with higher water retention capacity, moisture, and thickness [25]. Therefore, the good rheological property of starch–mucilage film is highly dependent on the synthesis method of films. However, this rheological property can be controlled by adding mucilage and starch in various concentrations. Meanwhile, extrusion or dry method of film formation is usually used at a large commercial scale. This method can improve the functional or physicochemical properties and chemical structure of starch–mucilage films [26]. Generally, this method is divided into three zones: the beginning part of the machine (feeding zone), mixing of the sample (kneading zone), and ending part from the machine (heating zone). In this regard, tubular films were prepared from *Opuntia ficus-indica* mucilage (10% *W*/*W*) with waxy maize and acetylated or normal rice starches (70% *W*/*W*) and glycerol (20% *W*/*W*). addition of *Opuntia ficus-indica* mucilage improved the functional and mechanical properties of tubular and extruded films [16]. Because of the interaction of mucilage and glycerol as a plasticizer and the partial breakdown of the starch structure during the extrusion process, it is less susceptible to acetylation. Moreover, polyvinyl alcohol (PVOH) is an important synthetic biodegradable polymer having excellent flexibility, tear, high strength, and gas barrier properties, although it has poor dimensional stability due to high moisture absorption [27]. Furthermore, as compared to other commercial polymers, it has a very high price. As a result, polysaccharides such as starch may be blended with renewable and abundant agro-resources to minimize production costs. Several studies have been reported to improve the compatibility of starch and polyvinyl alcohol such as fillers, cross-linking agents, compatibilizers, and plasticizers [28]. However, as most of these cross-linking agents are usually toxic, their potential use as biomaterials has been limited. To overcome these drawbacks, the mechanical properties and water resistance of starch and polyvinyl alcohol films must be improved using nontoxic functional additives and simple modification techniques. Comparative research was executed by Gomez-Aldapa et al. [29], and films were produced from potato starch (5% *W*/*V*) blended with polyvinyl alcohol (PVOH) (4% *W*/*V*). Therein, glycerol (25% *W*/*W*) was used as a plasticizer. The result of a study proved that films prepared blended with starch and PVOH are highly suitable and manageable for food packaging applications. This is because the incorporation of PVOH into potato starch enhanced the water absorption capacity, and improved mechanical and functional properties and gas permeability of the film. However, biodegradable films produced from potato starch and PVOH blends had a homogeneous appearance, with no obvious bubbles or phase separation, were transparent, and were easy to unmold. Satisfactory compatibility was observed during the processing in all the blended formulations. Moreover, bindings of hydrogen bonds between polysaccharide chains of starch–mucilage and glycerol were analyzed by Fourier-transform infrared spectroscopy (FTIR) analysis of films prepared from the potato husk starch and pear peel mucilage [25]. Hydrogen bonding interaction (Figure 2) is responsible for the interactions of biopolymers (starch–mucilage) with absorbed water molecules. However, mucilage consists of galacturonic acid, L-rhamnose, D-galactose, D-xylose, and L-arabinose. These carbohydrate molecules of mucilage have excellent potential to interact with other molecules such as starch (Amylose and Amylopectin) and glycerol due to their high foaming ability [30]. Furthermore, the alternative galactose or arabinose branches prevent intramolecular hydrogen bondings from forming, also maintaining the molecule in an extended state where it may interact with the amylose molecule in the system via non-covalent hydrogen bonds, resulting in a more extended conformation [31,32]. As a result, the degree of pseudo-plasticity can be increased. The creation of polymer complexes encouraged by the release of amylose and low molecular weight amylopectin during the processing may be responsible for the increase in the viscosity of the starch–polysaccharide system on cooling. Moreover, the viscosity may be increased with increasing sucrose concentration due to crosslinking between starch chain and sugar units [17].

## 3. Physicochemical Properties and Characterization of the Starch–Mucilage Film

**Figure 3:**
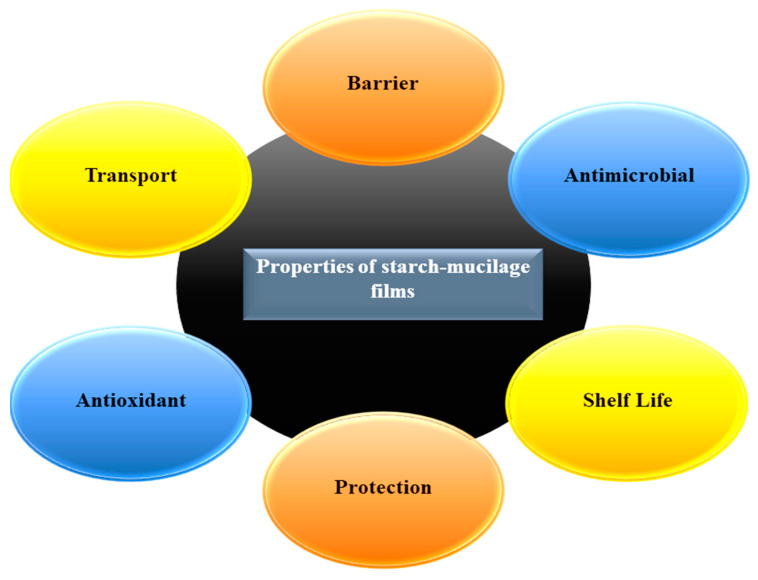
Major properties of starch–mucilage edible films.

Nowadays, physical and chemical modifications have been suggested as ways to improve the physicochemical and mechanical properties of starch–mucilage-based films, which seem to be very effective [33]. The major properties of starch–mucilage films are illustrated in Figure 3. Starch–mucilage edible films have several advantageous properties. They can enhance the shelf-life of food products, provide excellent protection against UV rays, barrier properties against mechanical damage (cuts and dents), retain the bioactive compounds of foods (antioxidants), and are helpful in the transport of solutes (pigments, additives, and salts). Moreover, desirable physicochemical properties and characterizations of starch–mucilage can be a favorable choice as an alternative to synthetic polymers, especially for food applications. Therefore, all these properties of films mainly depend on the extraction methods of starch–mucilage and synthesis of the film [17]. Several techniques are used for the evaluation of the characterizations of films such as: scanning electron microscopy (SEM), used for determining the surface morphology; Fourier-transform infrared spectroscopy (FTIR), used to evaluate the specific functional groups present in the sample; nucleus magnetic resonance (NMR), used for the determination of organic molecules; X-ray diffraction analysis (XRD), used to check the intensity of materials; thermogravimetry analysis (TGA), used for measuring the mass variations of a sample by temperature; and differential scanning calorimetry (DSC), used for the determination of physical and chemical changes during the thermal processing of a sample [3,4,34,35,36]. Furthermore, it has been observed that two or more polymers are commonly blended to obtain a wide range of biological and physicochemical properties of films. However, several properties of chitosan can be improved by blending with natural polymers such as starch, mucilage, and cellulose, as well as with synthetic ones such as graphene oxide, poly(vinyl pyrrolidone), poly(ethylene oxide), poly(lactic acid), zein, konjac glucomannan, and sodium alginate. Physicochemical properties including thermal stability, surface morphology, hydrophilicity, and hydrophobicity of blended films are mainly dependent on the types of biopolymers [37].

### 3.1. Fourier-Transform Infrared Spectroscopy (FTIR)

Fourier-transform infrared spectroscopy (FTIR) is a technique used to determine the functional groups and chemical structure of the film. Generally, it is used in the wavelength range between 4000 and 400 cm^−1^ [38]. In this context, Krystyjan et al. [24] characterized the film prepared from the *Plantago psyllium* starch–mucilage by FTIR spectroscopy. In their study, a high number of changes was observed in the spectrum of the film prepared from the starch only, compared to film prepared from the starch–mucilage film. Broadband spectra were recognized at about 3200–3300 cm^−1^ in O-H stretching. Furthermore, asymmetric vibrations of the ring appeared at around 1100 cm^−1^. Spectra at 1080–960 cm^−1^ corresponded to C-O groups with stretching vibrations. Spectra of all the prepared film samples presented bands at 3200 cm^−1^ and 2880–2900 cm^−1^ which confirmed the C-H group, as well as CH2 group, were confirmed bands at 1245, 1405–1465, 2855, and 2916–2936 cm^−1^. Similarly, in chia-seed mucilage nanocomposites, films with starch nanocrystals were produced [3], and the FTIR spectra of the broad absorption peak were recorded around 3290 cm^−1^ linked to the OH (hemicellulosic) group. Characteristic peaks of protein structures were observed at 1417, 1545, and 1643 cm^−1^. Spectra were deconvoluted at the range between 1200 and 900 cm^−1^. Moreover, the observed peak at 1033 cm^−1^ was due to hemicellulosic compound glycosidic linkage. Peaks of aliphatic CH wagging were detected around 922 cm^−1^. The OH groups in starch and glycerol, which are added to the mucilage film, are likely to create new functional groups with hydrogen bonds in the mucilage structure; therefore, the OH stretching band will become wider as the H bands increase due to the formation of new intermolecular hydrogen bonds. Likewise, Wang et al. [39] produced an edible film composed of *Dioscorea opposita Thunb*. mucilage (DOM) and starch. In their study, there were peaks at 1250 cm^−1^ when the effects of pure starch and DOM films are combined. Peaks at 1250 cm^−1^, 923 cm^−1^, and 525 cm^−1^ were seen in the spectra of *Dioscorea opposita Thunb*. mucilage films and starch only when the effects of pure mucilage, starch, and DOM films were combined. In another study, psyllium gum and modified starch composite films were prepared by Askari et al. [40]. In this study, films were examined for FTIR study. The characteristic peaks of starch were at 860–1250 cm^−1^ (C-H stretching and C-O stretching of the anhydroglucose ring), 1593 cm^−1^ (O-H blending of water absorbed), 2924 cm^−1^ (C-H stretching), and 3000–3600 cm^−1^ (O-H stretching). Moreover, increases in the percentage of *Psyllium* gum enhanced the intensity of the broad-band at 3300 cm^−1^ (O-H stretching of starch). This might be due to *Psyllium* gum having more hydroxyl groups, which could lead to more hydrogen bonding.

### 3.2. Scanning Electron Microscopy (SEM)

Scanning electron microscopy is a part of an electron microscope, used to evaluate the surface morphology of starch–mucilage films. Electrons are bombarded on the prepared sample through electron beams resulting in the reflection of electrons towards the object from the sample [41]. Generally, coating of film samples can be carried out with gold for evaluation of surface morphology. Moreover, swollen-dried and freeze-dried films were prepared from corn starch and okra mucilage [15]. The result of the study revealed that the structure of the dry film was free from cavities, and had a homogeneous appearance and flat structure, without major imperfections due to strong interaction between starch, mucilage, and glycerol. During the gelatinization (breakdown of starch bonds) heating of solution, these all were subjected. There are two zones of starch granules (crystalline zone and amorphous zone). In the case of the crystalline zone, hydrogen bonds can be broken down in the presence of heat [42]. Therefore, an amorphous zone causes the gelatinization of starch, and swelling of the amorphous zone develops. Moreover, kinetic energy was decreased after cooling the solution, which creates new interactions between molecules. However, during the drying of films, strong bonds were created between the okra mucilage structure and corn starch. To obtain stable and flexible films, a plasticizing agent was used for reinforcement. The thickness and microstructure of a film matrix are directly influenced by the surface and interior heterogeneity of the film matrix, according to Gutiérrez et al. [43]. As a result, it is clear that the more complicated and irregular the arrangement of molecules, the more likely it is that micrographs may have errors. Likewise, the film was prepared from the *Dioscorea opposita Thunb.* mucilage and starch [39], and also, the effects of sodium carboxymethyl cellulose (CMC) and ultrasound were observed by scanning electron microscopy. They prepared four samples with different compositions of starch, mucilage, glycerol, and sodium carboxymethyl cellulose. SEM results showed that dry films were free from imperfections or cracks, free from cavities, had clear starch granules, smooth structure, and was homogeneous. This property may be due to interactions between starch, mucilage, glycerol, and CMC during the process of gelatinization with heating. The hydrocolloids assemble themselves in the film matrices, and during heating, the hydrogen bonds may be broken, causing the crystalline zone to develop. The starch expanded during film production, producing granule expansion and the gelatinization of the starch [44]. Likewise, films were prepared from the gelatin of chicken skin and tapioca starch by Loo and Sarbon [45]. Scanning electron microscopy (SEM) micrographs confirmed that the blended films had an excellent smoother surface and improved internal structure over pure gelatin films. Moreover, the surface of gelatin films with 5 and 25% tapioca starch was observed to be enhanced by removing surface unevenness, resulting in smoother film surfaces. The changes in the surface and cross-sectional area of the prepared films were most likely caused by changes in the components of the blended films, as well as interactions that occurred in the film matrix as a result of the addition of tapioca starch. Chen et al. [46] investigated the physicochemical and mechanical characteristics of maize starch films containing cotton linter nano-cellulose, bamboo nano-cellulose, and sisal nano-cellulose. The SEM pictures revealed that it has the strongest reinforcing mechanical strength because bamboo nano-cellulose has the highest aspect ratio.

### 3.3. Thermal Stability of Films

Differential scanning calorimetry (DSC) and thermogravimetric analysis (TGA) can be used to determine the behavior of physical and chemical changes during the thermal processing, and the difference between the distinct formulation of starch–mucilage films, respectively [47]. Araujo et al. [15] prepared a film from the okra mucilage and corn starch. The okra mucilage precipitate’s differential scanning calorimetry curve showed loss of water from the material between 0 and 175 °C, and melting temperature of precipitated mucilage components with an endothermic peak was observed at 180 °C. Moreover, with the modification of pectin and comparing it with the DSC curve, an unusual endothermic peak was observed nearby 140 °C. Degradation of the films occurred at about 250 °C and endothermic peaks were reached at about 315 °C. However, DSC also confirmed that the high rate of mucilage degradation is due to the high degree of methoxylation of the carboxylic acids in pectin [48]. In the case of *Plantago psyllium* starch mucilage films, an endothermic effect was observed at low temperature with the relation of water polysaccharides [24]. Five films were evaluated for thermal analysis in different starch–mucilage concentrations. In their study, they observed that both film based on potato starch (H1) (3% *W*/*W*) and mucilage (0.1% *W*/W) and film based on potato starch (H2) (3% *W*/*W*) and mucilage (0.2% W/*W*) contained approximately 15% less water than film based on single potato starch (H0). The highest effect of exothermic with comparable magnitude was observed close to 330 °C. The thermal characteristics of S0 are different from those of S1 and S2 as determined by DSC. Its thermogram showed two exothermic peaks with maximum temperatures of 304 °C and 237 °C (which might be linked with decomposition), but S1 had just one peak at 291 °C and 317 °C which was connected with film composite degradation. This may be due to the addition of mucilage to the solution reducing decomposition, making the films more thermally stable. Furthermore, the addition of mucilage decreased the water content in the system based on the endothermic peak at around 70 °C, which corresponds to the dehydration of some film samples due to mucilage filling the gaps between starch molecules that were interacting with each other through hydrogen bonds. On the other hand, thermogravimetry analysis (TGA) is used for the measuring of mass variation of starch–mucilage films by temperature [49]. Edible films were prepared by *Dioscorea opposita Thunb.* mucilage and starch by Wang et al. [39]. Around 10% weight loss at 100C (indicating the water evaporation), and 48% weight loss at 300 to 350 °C were observed. Moreover, degradation of starch occurred at around 322 °C. Various studies revealed that the thermal property of mucilage is highly dependent upon the conformation of molecules, structures, and behavior of the materials. In this context, they observed continuous weight loss of *Dioscorea opposita Thunb.* mucilage films without apparent peaks in TG.

## 4. Mechanical and Physical Properties of Starch–Mucilage Films

### 4.1. Tensile Strength

Generally, the tensile strength of the film is the most important mechanical property of starch–mucilage films. Therefore, the casting method is widely used to obtain a high mechanical parameter of starch–mucilage films. Good tensile strength and low elongation are significant to provide the better structural protection of food products during the transportation and storage of products [50]. Because the branching amylopectin chains in solution (after being pasted in hot water) have poor ability to interact, the gels and films produced are weak. Due to linear chains of amylose having a high tendency to interact via hydrogen bonds, amylose gels and films are stiffer than amylopectin gels and films [22]. Moreover, the tensile strength of starch-based films is dependent upon the amylose and amylopectin ratio. The addition of starch into mucilage resulted in the reduction of the tensile strength of films. Several studies revealed that films prepared from the nanoparticles of starch incorporated with the mucilage showed a positive impact on the tensile strength. In a study, the film prepared from the nanoparticle’s potato starches (6%) showed increasing the tensile strength [5]. In another study carried out by Mujtaba et al. [3], films were prepared from the chia-seed starch nanocrystals and mucilage, and it was observed that the tensile strength of films was decreased in the case of 6% starch–mucilage nanocrystal and 3% mucilage–starch nanocrystals due to interactions between mucilage and starch components. Similarly, the tensile strength of okra mucilage and corn starch film (66.98 to 140.30 MPa), *Dioscorea opposita Thunb.* mucilage and starch films (0.79 to 4.26), *Plantago psyllium* seed mucilage and starch (0.56–1.38 MPa), chia-seed mucilage and starch nanocrystals films (6.7–7.5 MPa), nopal mucilage, and rice starch (2.80–3.96 MPa) were observed [15,39,51]. As compared to the tensile strength of thermoplastic starch and *Opuntia ficus indica* mucilage (0.64–3.75 MPa), mango kernel starch and guar and xanthan gums (3.57–10.24 MPa), banana starch and peel fibers (8.9–11.1 MPa), and potato starch and coconut fiber nanocrystals (4.09–8.20 MPa) were observed as shown in Table 1. The addition of xanthan gums and guar gums created a reinforcing effect on the tensile strength of composite films prepared from the mango kernel starch [52]. Tensile strength of films was increased significantly for xanthan gum from 7.35 to 8.70 MPa and from 8.62 to 10.24 MPa for guar gums at 20% and 10% respectively. Most likely, the variation trends of tensile strength were consistent with various starch–gum composites films that demonstrated excellent tensile strength as the concentration of added gums increased [53]. Due to the impossibility of polymeric chains moving to maintain the elastic behavior observed in the absence of gum, starch and gum chains created a more strong network through hydrogen bonds, but with lower deformability. Starch–gum interaction may have both hindered and prevented the amylose–amylose interaction. Xanthan and guar gums improved the TS of films but resulted in a less deformable matrix [54]. In addition, nano-cellulose is significantly used with the starch blend for food packaging applications. This is because nano-cellulose exhibited energetically active sites, better mechanical strength, high surface area, and high crystallinity. Due to these features, it has excellent potential in the biomedicine, pharmaceuticals, and food packaging industries [55]. Cellulose nanofibers were extracted from the sugar palm and blended into starch biocomposites from 0 to 1% concentration. The result showed an increase in the Young modulus (121.26 MPa) and tensile strength (4.8 MPa). Nanocrystals and cellulose nano-fibers exhibited excellent mechanical properties [56]. Three main factors affected the mechanical properties of nano-composite materials such as nanostructure of the matrix and matrix/filler interface, processing methods, and morphology and dimension. Nanocellulose with a specific surface area and high aspect ratio, as well as a rough surface and smaller fibers, can increase nanofiller/matrix adhesion and mechanical performance. Considering their size, cellulose nanocrystals surpass cellulose nanofibers in terms of mechanical properties [48].

### 4.2. Water Solubility and Water Vapor Transmission Rate (WVTR)

WVTR (water vapor transmission rate) and water solubility are key properties and parameters evaluated during the characterization of films that help us to understand how the film behaves and interacts with water. It can be also measured by a water vapor transmission tester [57]. The result of a study indicated that film prepared from the *Dioscorea opposita Thunb.* mucilage and starch can be used as an edible coating or edible films due to excellent water solubility—41.11% to 62.74% [39]. In addition, the ranges of water solubility of *Plantago psyllium* seed mucilage and starch (16.76–22.85%), okra mucilage and corn starch (11.53–89.82%), chia-seed mucilage and starch nanocrystals (80–86.1%), nopal mucilage and Rice starch (18.42–22.59%), prickly pear peel mucilage and potato husk starch (39.67–54.43%) were observed, as compared to mango kernel starch and guar and xanthan gums (36.26–44.63%), potato starch and zedo gums (25.02–43.67%), and cassava starch and hydroxyethyl cellulose (28.73–93.26%) [3,24,40,58,59,60,61,62]. The addition of starch with other biopolymer blends can improve the physicochemical and mechanical properties of films. For example, the viscosity of tapioca starch pastes was increased with the addition of guar gum and xanthan gum. Moreover, xanthan gum with tapioca starch blend exhibited lower peak viscosity than starch–guar gum blends [54]. Additionally, swelling power (SP) of cationic topica starch was sharply increased with increases in the temperature of the system (from 60 to 70 °C), while SP of starch and gums blend was decreased from 70 to 90 °C. Furthermore, water solubility and SP of the starch–gum blend resulted in the same as those of starch alone. Consequently, composites films were prepared from the mango kernel starch and guar–xanthan gums. The addition of xanthan gum and guar gum increased the solubility of films. Moreover, films were not disintegrated and also remained intact [52]. In the case of *Plantago psyllium* seed mucilage and starch, films were highly soluble in water as compared to alone starch-based films [24]. Moreover, the addition of mucilage into the starch blend increased the solubility of the film. The mechanical characteristics of a film are determined by its use; when it has low elongation, it is necessary to have a higher tensile strength, which ensures the product’s structural stability. It is believed that the high-water solubility of films is the most desirable element for food packaging applications because it can be easily removed from food coating applications. The high solubility of films is dependent upon the number of free hydroxyl groups in the mucilage [63]. Furthermore, water vapor permeability (WVP) of *Dioscorea opposita Thunb* film ranged from 51.85 ± 2.56 to 63.08 ± 1.07 g mm/m^2^ d kPa [39].

In another study carried out by Krystyjan et al. [24], 2 cm of film was used and they dried it at 105 °C for 24 h in a hot air oven. Consequently, the result showed that the addition of mucilage increased the solubility of films, and starch–mucilage films were more highly soluble in water than single starch films. It is also possible that low molecular mucilage extract components that were not included in the film network were readily removed from the film structure, resulting in increased water solubility of the films. The incorporation of gums into starch did not allow us to observe a significant change in the water vapor permeability (WVP) of mango kernel starch films [52]. The results proved a clear difference, with WVP values increasing significantly as the concentration of guar gum was increasing. Moreover, the results increased from 1.18 *×* 10^−10^ (control) to 4.29 *×* 10^−10^ with 0.6% guar gum. Starch-based edible films have a low barrier capacity against water vapors due to their hydrophilic nature [64]. Furthermore, the hydrophilic property of both guar and xanthan gum attracts water molecules, resulting in mobile areas with longer interchain lengths. Additionally, these hydrophilic gums compete with water at the polymer matrix’s active sites, causing water clustering and micro-cavities in the network structure [65].

### 4.3. Transparency and Thickness of the Film

Transparency is one of the most important aspects of packaging material, especially when product visibility is important. In such cases, the package’s degree of transparency is a critical characteristic that might influence a consumer’s choice to buy a product [66] a product [66]. The transparency of any component is a sign of the degree to which light is permitted to pass through it. Moreover, the incorporation of mucilage into starch contributes to a major statistically significant difference in the transparency of films. Similarly, transparency of starch–mucilage nanocomposites films was recorded as 6% starch nanocrystals and mucilage (42.6 ± 0.57 μm), 3% starch nanocrystals and mucilage (42.6 ± 0.57 μm) and for control (45.6 ± 0.57 μm). The thickness result of nano starch and chia-seed mucilage showed that an increase in starch nanocrystal concentration in mucilage decreased the thickness of the film, while there was no significant effect on the overall thickness of films when increasing the concentration of starch nanocrystals. These can be attributed to good film composite formation between the starch nanocrystals and mucilage [3]. Consequently, varying amounts of starch formulations could not influence the thickness measurements of the film prepared from the okra mucilage and corn starch [15]. Mohammadi et al. [67] also observed the zinc oxide nanoparticles and okra mucilage in the films, which showed the highest thickness. Furthermore, the thickness of several binary biopolymer-based films was observed such as *Dioscorea opposita Thunb*. mucilage and starch (0.21–0.34 mm), *Plantago* *psyllium* seed mucilage and starch (0.130–0.209 mm), okra mucilage and corn starch (0.04–0.08 mm), chia-seed mucilage and starch nanocrystals (0.042–0.045 mm), nopal mucilage and rice starch (0.47–0.50 mm), and prickly pear peel mucilage and potato husk starch (0.09–0.22 mm) were observed as compared to thermoplastic starch and *Opuntia ficus indica* mucilage (0.125–0.150 mm), potato starch and zedo gums (0.22–0.207 mm), and cassava starch and hydroxyethyl cellulose (0.04–0.08 mm) [3,15,24,25,58,59,60,61,62]. Pea starch-based film-forming solution with varying concentrations of glycerol and guar gum produced transparent and uniform films. Pea starch showed a significant positive linear effect on transparency, whereas guar gum and glycerol showed a significant negative effect. The highest transparency value of films was observed at a high concentration of pea starch. When the glycerol and guar gum concentrations were raised (*p* < 0.05), the transparency of the films decreased, most likely due to greater polymeric chain compaction changing the refractive index and restricting light passage through the film matrix [68].

### 4.4. Antimicrobial Activity and Bio-Degradation of Film

**Figure 4:**
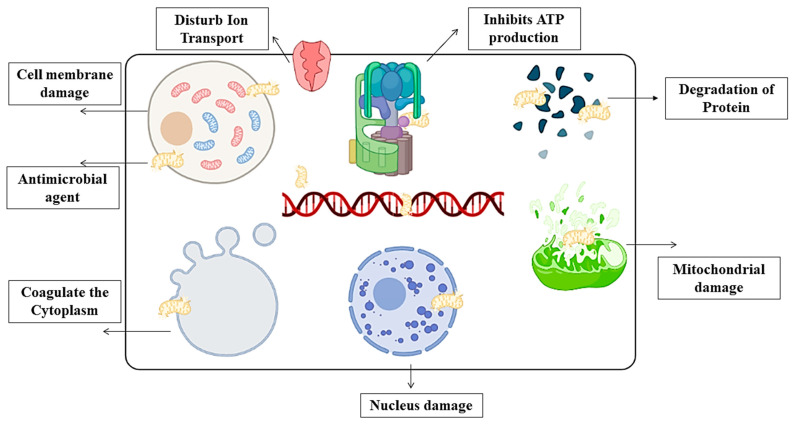
The scheme represents the proposed antimicrobial mechanisms of biopolymer blended film.

Natural gums such as mucilage are biocompatible, environmentally friendly, and capable of a wide range of chemical changes. However, due to their low water solubility and distinct flavor, their industrial applicability is limited. Composite technology can help improve solubility and stability [69,70]. The antimicrobial activity of binary film highly depends on the antimicrobial agents, which can be classified based on their origins such as synthetic antimicrobial agents and natural antimicrobial agents [38]. However, naturally originating antimicrobial agents are regarded as safe for human consumption and easy to obtain. Moreover, various essential oils are also incorporated with starch–mucilage for enhancement of their antimicrobial ability [71]. Moreover, films prepared from starch and mucilage have excellent antimicrobial activity against Gram-positive and Gram-negative bacterias. Therefore, they can prevent the food from the various pathogenic bacterias and have excellent mechanical properties such as a barrier to moisture and oxygen, stiffness, high tensile strength, and flexibility [34]. Furthermore, the crystalline region of starch can produce a parclose against several gases and microorganisms, and the effect becomes stronger for high amylose-containing films than for amylopectin films. In this context, electrostatic interaction between positively charged chitosan and bacteria with negatively charged cellular membranes (such as certain types of coliform bacteria and aerobic mesophilic bacteria) changes the barrier characteristics of these membranes significantly [65]. This includes changing the flow of nutrients and waste, resulting in the bacterium’s destruction. However, the phospholipids of Gram-negative bacteria’s cellular membranes interact with the NH-groups in chitosan, resulting in the bacteria losing cellular material. The chelating ability of chitosan can have an impact on microbial growth [29]. Starch and mucilage films can attach to the Gram-positive and Gram-negative bacterial cell walls through many interactions in the bacterial cell membrane. Therefore, the viability of bacterial cells can be reduced by inactivating the DNA replication, and also several antimicrobial agents such as essential oils and nanocarriers (nanoparticles, nanocomposites, nanocrystals, and nanofibers) can be incorporated with starch–mucilage films. These antimicrobial agents can play a very significant role in the incorporation of starch and mucilage. They enter into the cell wall of bacteria and rupture the cell membrane, degrade the protein, damage mitochondrial and inhibit ATP production resulting ing in killing the bacteria as explained in Figure 4. [72]. The antimicrobial activity of chia-seed mucilage and nanocrystal starch was evaluated by Mujtaba et al. [3]. It was observed that the concentration of starch nanocrystal matter in the antimicrobial activity of films such as 6% starch nanocrystal and chia-seed mucilage has higher antimicrobial activity than 3% nanocrystal and mucilage. The antimicrobial experiment of the film was conducted again with several Gram-positive and Gram-negative bacterias including *B. thuringiensis*, *S. aureus*, *E. coli*, *S. typhmurium*, *P. aeruginosa* and *S. mutans*. The results of the antimicrobial assay were 18.23 ± 0.68, 18.89 ± 0.72, 19.56 ± 0.79, 17.86 ± 0.62, 18.19 ± 0.61, and 17.52 ± 0.51 mm, respectively. Moreover, several biopolymers have been investigated to be used in the development of biodegradable, edible, and antibacterial food packaging films, such as starch, mucilage, gums, cellulose, and lipids. Several researchers have studied chitosan/starch blends films. The incorporation of chitosan into corn starch increased the antimicrobial activity of films. In this context, biodegradable composite films were produced from brown rice starch (BRS) and chitosan (CH) by Hasan et al. [73]. In this study, the authors evaluated the antimicrobial activity of films against Gram-positive (*Staphylococcus auerues*) and Gram-negative bacteria (*Escherichia coli*). The inhibition zones of *Escherichia coli* against BRS100, BRS70CH30, BRS50CH50, BRS30CH70, and CH100 were 7.33 ± 0.33, 8.00 ± 0.00, 8.00 ± 0.00, 8.00 ± 0.00, and 8.00 ± 1.00, and for *Staphylococcus auerues,* they were 6.67 ± 0.58, 7.67 ± 1.15, 7.00 ± 1.00, 10.33 ± 0.58, and 12.33 ± 0.58, respectively. Chitosan’s antimicrobial activity is influenced by electrostatic interaction between the positive charge of the protonated amino group from chitosan and the negative charge on the surface of the microbial cell, resulting in the formation of a membrane on the microbe’s surface that prevents the microbe from gaining nutrients [69].

In addition, naturally derived biopolymers are widely accepted in the food packaging industry due to their high biodegradability [18]. To measure soil degradation rates of chia-seed mucilage and starch nanocrystal-based films, the films were incubated in soil for 15 days by Mujtaba et al. [3]. The loss of weight of the films is regarded as a sign of their deterioration in the soil in a soil degradation study. Water activity increases microorganism growth in films with high water absorption, and microorganisms that create enzymes in the soil also break down edible films. Based on the results of this study, it was concluded that all of the films were destroyed within 15 days. Nature-friendly materials such as 3% starch nanocrystalline and 6% mucilage films can be degraded easily in nature. In addition, glycerol, which is used as a plasticizer in edible films, was also observed to be metabolized by soil and degraded, resulting in mass loss.

## 5. Techno-Economic Challenges of Starch–Mucilage Films

Biodegradable polymers such as starch are some of the most important groups of commercially accessible bio-based products at the moment. Since the 1980s, a growing number of starch polymers have been developed. Thus, simple products like pure thermoplastic starch and polyolefin/starch blends were introduced in the beginning. These products had a detrimental influence on public perception of biodegradable polymers and harmed the reputation of the firms involved due to the incomplete biodegradability of starch/polyolefin blends [74]. However, modified starch polymers are presently the most widely used application, accounting for 75% of the overall market share for starch polymers. Moreover, starch–mucilage blends are significantly used in applications including a biodegradable film for wrap films, toys, technical films, and shopping bags [32]. Moreover, starch–mucilage polymers have a high-water vapor permeability, which is beneficial in applications like moisture-free packaging of hot meals. In addition to technical advancements toward biodegradable packaging, international and national government regulations have a role in the environmental problem of plastic accumulation. This is because there are several schemes and policies for reducing the realize of plastics in wastewater treatment plants [75]. Furthermore, several government agencies have implemented control measures such as advertising limitations, consumption regulation, taxes and fees to restrict the use of plastic, and the prohibition of single-use items. In addition, corporations have launched voluntary corporate social responsibility (CSR) initiatives to operate sustainably in environmental domains. As a result, synergy is required between government policies, industry voluntary activities, and changes in consumer behavior [76,77,78,79].

## 6. Future Research Perspectives and Conclusions

Currently, researchers are more focused on research to replace petroleum-based component plastics with plant-derived biopolymers, offering desirable properties. Additionally, due to the high impact of plastic-based materials on the environment, human health concerns for the formulation of biobased, non-toxic, biocompatible, and biodegradable polymer films are emerging. Several plant-derived biopolymers such as starch, cellulose, gums, agar, milk, cereal, and legume proteins are always the first choice for eco-friendly packaging materials as the substitute for non-biodegradable packaging materials. Starches from various sources have usually been considered as a favorite choice of material for food packaging applications, however, suitable barrier properties are impossible to attain with single polymeric packaging material. Therefore, the properties of edible films can be modified based on the hydrophobic–hydrophilic qualities of biomolecules. In certain chemical modifications of starch, however, the chemical residues may impart toxicity in the food commodity. Therefore, in such cases, several plant-derived polymeric combinations could be used as an effective binary blend of the polymer to improve the mechanical and barrier properties of packaging film. Recently, scientists have shown great interest in underutilized plant-derived mucilage to synthesize biodegradable packaging material with desirable properties. Mucilage has a great potential to produce a stable polymeric network that confines the starch granules, delaying the release of amylose and improving the mechanical property of films. Due to techno-economical challenges and lack of awareness, the utilization of biodegradable materials is still not mainstream. Therefore, more research is required to synthesize effective packaging materials with desirable properties.

## Figures and Tables

**Table 1:** Mechanical properties of various biopolymer blended films.

Source of Binary Blended Films	Thickness (mm)	Tensile Strength (MPa)	WVP	Elongation (%)	Water Solubility (%)	References
*Dioscorea opposita Thunb*. mucilage and starch	0.21–0.34	0.79–4.26	51.85–63.08 (g mm/m^2^ d kPa)	47.52–153.26	41.11–62.74	[40]
*Plantago psyllium* seed mucilage and starch	0.130–0.209	0.56–1.38	-	7.5–15.7	16.76–22.85	[24]
Okra Mucilage and Corn Starch	0.04–0.08	66.98–140.30	1.32–2.42 (g/m s Pa)	5.91–5.99	11.53–89.82	[15]
Chia-seed mucilage and starch nanocrystals	0.042–0.045	6.7–7.5	-	14–19	80–86.1	[3]
Nopal mucilage and Rice starch	0.47–0.50	2.80–3.96	0.21–3.10 (g mm m^−2^ h^−1^ kPa)	12.07–2.63	18.42–22.59	[16]
Prickly pear peel mucilage and Potato husk starch	0.09–0.22	-		-	39.67–54.43	[25]
Thermoplastic starch and *Opuntia ficus indica* mucilage	0.125–0.150	0.64–3.75	-	-	-	[41]
Mango kernel starch and guar and xanthan gums	-	3.57–10.24	1.28–4.29 × 10^−10^ (g m^−1^ s^−1^ Pa^−1^)	6.28–17.78	36.26–44.63	[52]
Potato starch and zedo gums	0.22–0.207	-	5.58–9.53 × 10^−11^ (g/m s Pa)	-	25.02–43.67	[58]
Banana starch and peel fibers	-	8.9–11.1	8.9–25.2 × 10^−11^ (g/m s Pa)	20.7–25.9	-	[59]
Corn starch and cellulose nanofibers	-	21.90–28–87	3.00–4.73 × 10^−7^ (g Pa^−1^ h^−1^ m^−1^)	73.07–103.80	-	[60]
Potato starch and coconut fiber nanocrystals	-	4.09–8.20		23.71–30.40	-	[61]
Cassava starch and hydroxyethyl cellulose	0.04–0.08	-	12.84–18.94 (g h^−1^ m^−2^)	-	28.73–93.26	[62]

## Data Availability

Data sharing does not apply to this article.
